# Acute Fulminant Cerebral Edema Caused by Coxsackievirus A6 Infection: A Case Report

**DOI:** 10.1002/ccr3.72101

**Published:** 2026-02-25

**Authors:** Kang An, Juan Qian

**Affiliations:** ^1^ Department of Hematology/Oncology Shanghai Children's Medical Center, Shanghai Jiao Tong University School of Medicine Shanghai China

**Keywords:** coxsackievirus A6, fulminant cerebral edema, hand, foot, and mouth disease, pediatric

## Abstract

In recent years, Coxsackievirus A6 (CV‐A6) has gradually replaced Enterovirus 71 (EV‐71) and Coxsackievirus A16 (CV‐A16) as the main pathogen causing hand foot mouth disease (HFMD) in China. This article reports a fatal case of HFMD caused by CV‐A6, leading to fulminant cerebral edema and cardiopulmonary arrest. A 6‐year‐old boy was admitted with a chief complaint of “fever for 1 day, two episodes of seizures”. On admission, the patient exhibited unresponsiveness, no spontaneous breathing, bilateral fixed and dilated pupils, complete muscle weakness, and loss of muscle tone. The brain computed tomography (CT) revealed diffuse decrease in brain parenchymal density. On the 3rd day of admission, the patient presented with a red rash on the hands, feet, knees, buttocks, and perianal area, and a nasopharyngeal swab was positive for CV‐A6. Considering the symptoms, physical examination, laboratory tests, and imaging results, the diagnosis of “severe HFMD, fulminant cerebral edema” is considered. On the 16th day of admission, the patient was diagnosed with brain death, and on the 57th day, the patient died. CV‐A6 infection can lead to severe neurological complications, characterized by fulminant cerebral edema, which can result in fatal consequences.

## Key Clinical Message

In recent years, coxsackievirus (CV)‐A6 has gradually replaced enterovirus 71 and CV‐A16 as the primary pathogen causing hand, foot, and mouth disease. The majority of CV‐A6 infections are mild cases, but a very small number of patients with CV‐A6 infection can develop acute fulminant cerebral edema, which can have fatal consequences.

## 1 Introduction

Hand, foot, and mouth disease (HFMD) is a common infectious disease among children, predominantly affecting those under the age of 5 years. The primary etiological agents responsible for HFMD include enterovirus 71, coxsackievirus (CV)‐A16, and others. In recent years, CV‐A6 has gradually replaced enterovirus 71 and CV‐A16 as the main pathogen causing HFMD in China [1, 2, 3].

The main symptoms and signs caused by CV‐A6 infection include fever, rash, peeling, and desquamation [3]. This article reports a case of acute fulminant cerebral edema in a 6‐year‐old male following CV‐A6 infection. After a literature review, a similar case of a 4‐year‐old female in Japan was found to have been previously reported [4]. Both patients were previously healthy children who developed cerebral edema after CV‐A6 infection ultimately leading to death, which is exceedingly rare.

The cause of acute fulminant cerebral edema caused by CV‐A6 infection is currently unclear. We aimed to analyze and summarize the clinical manifestations, laboratory findings, and imaging characteristics of this case as well as the literature review cases to raise awareness among clinicians, particularly in regions and seasons where enterovirus outbreaks are prevalent.

## 2 Case Presentation

A 6‐year‐old boy presented to the Emergency Department with a complaint of fever for 1 day and two episodes of seizures. The patient developed a fever 1 day before admission, with a maximum temperature of 39°C. Ibuprofen was used to reduce the fever. Six hours after the fever started, the patient experienced a seizure characterized by staring eyes and unresponsiveness, which lasted for several seconds before subsiding. There was no stiffness in the limbs, no urinary incontinence, and no cyanosis of the lips. Thirty minutes before admission, the patient came to our hospital due to a high fever. While waiting in the emergency department, the patient had a second seizure, which presented as a generalized convulsion. The patient was immediately taken into the resuscitation room for emergency treatment. Cardiac monitoring was initiated, oxygen was administered via face mask, and intramuscular midazolam was administered. Subsequently, the child developed cardiac and respiratory arrest, prompting immediate cardiopulmonary resuscitation and administration of adrenaline. After 15 min, the child regained spontaneous sinus rhythm. The child had a febrile seizure at the age of 2 years. The child was in good health with normal growth and development. There was no reported family history of neurological disorders.

The child was comatose and receiving tracheal intubation with positive pressure ventilation via a balloon catheter. The heart sounds were dull but regular, with coarse wet rales in the posterior lungs. The abdomen was flat without palpable liver or spleen. The Glasgow Coma Scale score was 3 (E1V1M1, range: 3–15). The pupils were dilated and fixed at 5 mm and unresponsive to light. Brudzinski's sign and Kernig's sign were negative. The patient had no physiological reflexes, including corneal, abdominal, patellar, and Achilles reflexes. Babinski's sign and Oppenheim's sign were also negative. The extremities were cold, and capillary refill time was 3 s.

## 3 Methods

**FIGURE 1:**
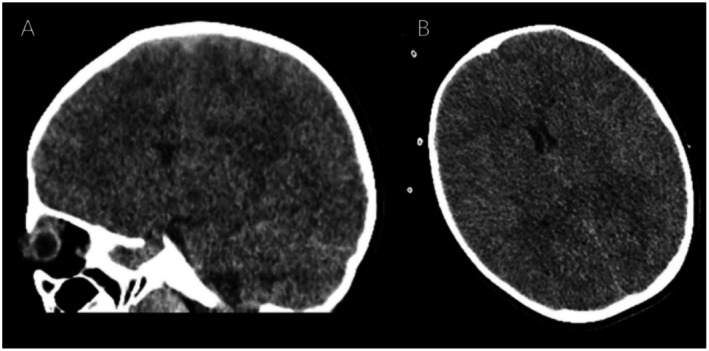
Cranial computed tomography showing a diffuse decrease in brain parenchymal density. (A) Sagittal view; (B) Transverse view.

**FIGURE 2:**
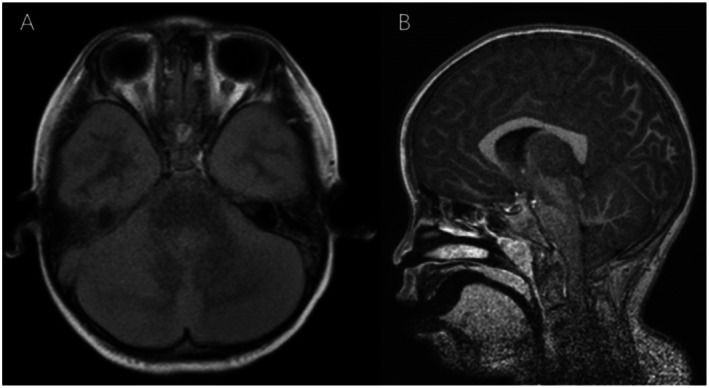
Cranial magnetic resonance imaging. (A) Magnetic resonance imaging revealed widespread shallow cerebral sulci, indistinct gray‐white matter differentiation, generalized abnormal signal intensity in the cerebral and cerebellar parenchyma, with low signal on T1‐weighted images, high signal on T2‐weighted images, high signal on fluid attenuated inversion recovery images, and high signal on diffusion‐weighted imaging; (B) There was displacement of the cerebellar tonsils laterally located below the foramen magnum and becoming pointed. The lowest point is at the level of the C3 vertebral body.

The laboratory test results of the patient are shown in Table 1. On the day of admission, cranial computed tomography revealed a diffuse decrease in the density of the brain parenchyma and significant cerebral edema (Figure 1). On the third day of hospitalization, cranial magnetic resonance imaging revealed tonsillar herniation (Figure 2). On the third day of hospitalization, the patient presented with a red rash on the hands, feet, knees, buttocks, and perianal area. Nasopharyngeal swab PCR testing confirmed the presence of CV‐A6 infection. Combined with the patient's medical history, the final diagnosis was severe HFMD, CV‐A6 infection, and fulminant cerebral edema.

**TABLE 1:** Laboratory test results.

Laboratory test	Result	Normal value
WBCs, billion/L	19.80	4–10
Neutrophils, %	81.8	33–74
RBCs, trillion/L	4.87	4.5–6.2
HGB, g/dL	128	131–179
PLT, billion/L	276	100–550
CRP, mg/L	18	0–8
TBIL, μmol/L	5.5	3–22
DBIL, μmol/L	0	0–5
ALT, U/L	185	< 50
AST, U/L	253	15–46
BUN, mmol/L	3.8	3.2–7.1
Cr, μmol/L	55	58–110
cTnI, μg/L	2.38	< 0.06
NT‐proBNP, pg/mL	247	0–125
IL‐6, pg/mL	95.45	1.05–15.80
Influenza A	Negative	Negative
Influenza B	Negative	Negative

Abbreviations: ALT, alanine transaminase; AST, aspartate transaminase; BUN, blood urea nitrogen; Cr, creatinine; CRP, C‐reactive protein; cTnI, cardiac troponin I; DBIL, direct bilirubin; HGB, hemoglobin; IL‐6, interleukin 6; NT‐proBNP, N‐terminal pro B‐type natriuretic peptide; PLT, platelets; RBC, red blood cell; TBIL, total bilirubin; WBC, white blood cell.

## 4 Results

After admission, the patient was given mannitol to lower intracranial pressure. Fentanyl and midazolam were administered for pain relief and sedation. Additionally, measures for hypothermic brain protection were initiated. On the second day of hospitalization, the patient developed central diabetes insipidus, which was managed symptomatically. On the 16th day of admission, the patient was diagnosed with brain death. The patient died on the 57th day after admission.

## 5 Discussion

CV‐A6 is a single‐stranded RNA virus belonging to the Picornaviridae family and Enterovirus genus and is primarily transmitted through close contact and respiratory droplets [5]. CV‐A6 infection presenting initially with acute fulminant cerebral edema is extremely rare, with only 1 case [4] besides the present case reported in the literature. The characteristics of these 2 cases are summarized in Table 2. Both patients were previously healthy individuals with no underlying diseases. Each patient experienced the onset with high fever that was followed by seizures and the rapid occurrence of cardiac arrest. Severe cerebral edema was observed by brain CT of both patients. In the present case, the brain MRI revealed cerebral edema and tonsillar herniation, and it was postulated that tonsillar herniation might have directly contributed to the cardiac arrest. IL‐6 is a multifunctional cytokine that plays a critical role in various physiological and pathological processes. It is produced by a variety of cell types, including immune cells, fibroblasts, and endothelial cells [6, 7]. Excessive levels of IL‐6 can lead to tissue damage, organ failure, and even death. Both the patient in this case and the Japanese patient showed elevated levels of IL‐6, which we consider to be related to CV‐A6 infection. Monitoring the levels of IL‐6 dynamically in clinical practice can help understand the changes in the infection status of pediatric patients.

**TABLE 2:** Clinical, imaging, and laboratory characteristics of the patients.

Case	1	2
Age (years)	4	6
Sex	Female	Male
Related viruses	Throat swab positive for CV‐A6 and stool swab positive for CV‐A6	Nasopharyngeal swab positive for CV‐A6
Initial symptoms and signs	Day 1: Fever of 39°C; Day 4: Rash on the face and trunk; Day 5: Generalized stiffness and spasms, cardiac arrest, respiratory arrest, dilated and fixed pupils, loss of corneal reflex and pupillary light reflex	Day 1: Fever of 39°C, generalized stiffness and spasms; Day 2: Generalized stiffness and spasms, cardiac arrest, respiratory arrest, dilated and fixed pupils, loss of corneal reflex and pupillary light reflex; Day 3: Rash on the hands, feet, and buttocks; Day 16: Brain death
Initial laboratory data	WBC 11300/μL, CRP 0.3 mg/dL; Cerebrospinal fluid: NA; Liver and kidney function: Normal; IL‐6: 32.1 pg/mL	WBC 19.80 × 10^9^/L, HGB 128.0 g/L, PLT 276 × 10^9^/L; Cerebrospinal fluid: NA; Liver and kidney function: Mildly elevated ALT and AST, normal for the rest; IL‐6: 95.45 pg/mL
Imaging	Brain CT: Marked cerebral edema	Brain CT: Diffuse decrease in brain parenchymal density, cerebral edema; Brain MRI: Cerebral edema, tonsillar herniation
EEG	No electrical activity	No electrical activity
Course of illness(days)	21	57
Outcome	Death	Death

Abbreviations: ALT, Alanine transaminase; AST, Aspartate transaminase; CRP, C‐reactive protein; CT, Computed tomography; CV‐A6, Coxsackievirus A6; EEG, Electroencephalogram; HGB, Hemoglobin; IL‐6, Interleukin 6; MRI, Magnetic resonance imaging; NA, Not available; PLT, Platelet; WBC, White blood cell.

Recently, CV‐A6 has emerged as the primary pathogen causing HFMD in China. Zhao et al. analyzed relevant epidemiological data [8]. They found that annual outbreaks have been caused by CV‐A6 since 2013. CV‐A6 predominantly causes mild cases, and severe cases accounted for 0.1% [95% confidence interval (CI): 0%–0.2%]. Currently, research on risk factors associated with severe CV‐A6 infection is limited and primarily focuses on genetic and clinical factors. Meng et al. analyzed the genetic susceptibility of Han Chinese children with severe and mild HFMD caused by CV‐A6 infection [9]. The analysis revealed that the single nucleotide polymorphism (commonly known as SNP) rs10879355 was associated with severe CV‐A6 HFMD. The CC genotype had a higher risk of severe infection compared to the TT or TC genotypes [odds ratio (OR): 2.48; 95% CI: 1.34–4.56]. Additionally, the SNPs rs4290270 and rs10879355 were found to be in complete linkage disequilibrium.

Another study conducted by Li et al. [10] indicated that the presence of birth complications (OR: 4.18; 95% CI: 1.64–10.63), peak body temperature exceeding 39°C during the course of illness (OR: 4.04; 95% CI: 2.29–7.10), and being the first‐born child (OR: 2.17; 95% CI: 1.27–3.70) increased the risk of severe HFMD in children. On the other hand, breastfeeding (OR: 0.52; 95% CI: 0.32–0.87) and handwashing after play (OR: 0.58; 95% CI: 0.34–0.97) showed a negative correlation with the severity of the disease.

In this study, our patient was presented with acute cerebral edema caused by CV‐A6. There is an increasing number of large‐scale studies on pediatric acute cerebral edema. A study from Taiwan indicated that the incidence of acute cerebral edema among patients diagnosed with acute encephalitis was 2.4% (25/1038), with 48% of the patients in the age group of 5–8 years, and the mortality rate was 65%. Survivors all had neurological sequelae. The neurological symptoms of pediatric acute cerebral edema patients included altered consciousness, vomiting, and headache; among those, 76% of patients had seizures 24–48 h before the onset of severe signs of cerebral edema. Signs of brain herniation appeared 0–9 days after the appearance of neurological symptoms [11]. Another study from the United States revealed that patients diagnosed with acute encephalitis had an incidence of acute cerebral edema of 1.5% (30/1955), with a median age of 8.2 years (ranging from 1 to 18 years) and a high mortality rate of 80%. In comparison to non‐fulminant cerebral edema cases (14%), there was a higher proportion of individuals of Pacific Islander descent in the fulminant cerebral edema cases (44%, *p* < 0.01) [12]. Unlike the previous two studies, Nukui et al. proposed that acute cerebral edema is a new subtype of acute encephalopathy [13].

Considering the child's symptoms and signs of intracranial hypertension at the time, along with the imaging findings of diffuse cerebral edema and brain herniation, to avoid exacerbating the brain herniation with lumbar puncture, we did not perform lumbar puncture and cerebrospinal fluid testing on the child. We are also considering the most appropriate timing for lumbar puncture and cerebrospinal fluid examination in similar patients in the future, to avoid worsening the patient's condition. Due to the lack of cerebrospinal fluid testing in both this case and the reviewed case, combined with the patients' clinical presentation, laboratory data, and imaging characteristics, these two patients should be classified as having acute encephalopathy caused by viral infection.

Based on the findings of previous studies, we believe that fulminant cerebral edema, whether classified as a subtype of acute encephalitis or a subtype of acute encephalopathy, deserves significant attention due to its unique clinical course and radiographic characteristics. Prospective studies related to fulminant cerebral edema, particularly more aggressive surgical interventions such as ventricular drainage and decompressive craniectomy, warrant further exploration.

The early identification of fulminant cerebral edema and proactive intervention for reducing intracranial pressure is crucial for decreasing mortality. We suggest that clinicians in areas where enteroviruses are prevalent should suspect enterovirus infection when children present with severe neurological symptoms, regardless of the presence of typical skin and mucosal manifestations.

## 6 Conclusion

CV‐A6 infection can lead to severe neurological complications characterized by fulminant cerebral edema, which can be fatal.

## Author Contributions

**Kang An:** data curation, methodology, writing – original draft. **Juan Qian:** conceptualization, supervision, validation.

## Funding

The authors have nothing to report.

## Consent

Verbal and written consent was obtained from the patient to publish this case.

## Conflicts of Interest

The authors declare no conflicts of interest.

## Data Availability

All data underlying the results are available as part of the article and no additional source data are required.
